# Vascular Endothelial Growth Factor-Receptor 1 Inhibition Aggravates Diabetic Nephropathy through eNOS Signaling Pathway in *db/db* Mice

**DOI:** 10.1371/journal.pone.0094540

**Published:** 2014-04-23

**Authors:** Keun Suk Yang, Ji Hee Lim, Tae Woo Kim, Min Young Kim, Yaeni Kim, Sungjin Chung, Seok Joon Shin, Beom Soon Choi, Hyung Wook Kim, Yong-Soo Kim, Yoon Sik Chang, Hye Won Kim, Cheol Whee Park

**Affiliations:** 1 Seoul St. Mary's Hospital, Department of Internal Medicine, College of Medicine, the Catholic University of Korea, Seoul, Korea; 2 Bucheon St. Mary's Hospital, Department of Rehabilitation Medicine, College of Medicine, the Catholic University of Korea, Bucheon City, Korea; National Center for Scientific Research Demokritos, Greece

## Abstract

The manipulation of vascular endothelial growth factor (VEGF)-receptors (VEGFRs) in diabetic nephropathy is as controversial as issue as ever. It is known to be VEGF-A and VEGFR2 that regulate most of the cellular actions of VEGF in experimental diabetic nephropathy. On the other hand, such factors as VEGF-A, -B and placenta growth factor bind to VEGFR1 with high affinity. Such notion instigated us to investigate on whether selective VEGFR1 inhibition with GNQWFI hexamer aggravates the progression of diabetic nephropathy in *db/db* mice.

While diabetes suppressed VEGFR1, it did increase VEGFR2 expressions in the glomerulus. *Db/db* mice with VEGFR1 inhibition showed more prominent features with respect to, albuminuria, mesangial matrix expansion, inflammatory cell infiltration and greater numbers of apoptotic cells in the glomerulus, and oxidative stress than that of control *db/db* mice. All these changes were related to the suppression of diabetes-induced increases in PI3K activity and Akt phosphorylation as well as the aggravation of endothelial dysfunction associated with the inactivation of FoxO3a and eNOS-NOx. In cultured human glomerular endothelial cells (HGECs), high-glucose media with VEGFR1 inhibition induced more apoptotic cells and oxidative stress than did high-glucose media alone, which were associated with the suppression of PI3K-Akt phosphorylation, independently of the activation of AMP-activated protein kinase, and inactivation of FoxO3a and eNOS-NOx pathway. In addition, transfection with VEGFR1 siRNA in HGECs also suppressed PI3K-Akt-eNOS signaling.

In conclusion, the specific blockade of VEGFR1 with GNQWFI caused severe renal injury related to profound suppression of the PI3K-Akt, FoxO3a and eNOS-NOx pathway, giving rise to the oxidative stress-induced apoptosis of glomerular cells in type 2 diabetic nephropathy.

## Introduction

The most pathognomonic pathologic finding of diabetic nephropathy, ‘Kimmelstiel-Wilson lesion’, is a nodular sclerosis that results from microaneurysmal dilatation of glomerular capillaries and mesangiolysis. Newly developed angiogenesis and abnormal capillaries found in the vicinity of the glomerulus are characteristics of early stage of diabetic nephropathy [1]. Among the various angiogenic molecules, vascular endothelial growth factor (VEGF) and angiopoietins are the main factors that have been linked to diabetic nephropathy [2].

VEGF-A, a potent inducer of vasopermeabiltiy and angiogenesis, contributes to glomerular capillary hyperpermeabilty of macromolecules that underlies the pathogenesis of diabetic nephropathy [3], [4]. VEGF-A acts mainly through two receptor tyrosine kinases; VEGFR1 (Flt-1) and VEGFR2 (Flk-1/KDR). In normal kidneys, VEGF-A is predominantly expressed in glomerular podocytes. However, while both VEGFR1 and VEGFR2 are expressed mainly in the glomerular endothelial cells [5], whereas conditioned human podocytes express only VEGFR2 and VEGFR3 but not VEGFR1 [6]. Increased VEGF-A and VEGFR2 expressions are exhibited in the rodent models of type 1 and 2 diabetic nephropathy. In early stage of diabetic nephropathy, VEGF-A acts in a novel autocrine and paracrine signaling mode to induce diabetic nephropathy [3]. Abundant experimental results demonstrate that VEGFR2 is the primary functional receptor that transduces both angiogenic and vascular permeability signals, whereas the function of VEGFR1 is less well pronounced. Recent studies suggested that one of main functions of VEGFR1 is to act as a decoy receptor, sequestering VEGF from binding to VEGFR2, especially as a secreted form (sFlt1) [7], [8]. Systemic overexpression of sFlt1 with adeno-associated virus-1, is associated with decreased amount of albuminuria and severer form of tubulointerstitial disease through the interruption in the binding of VEGF-A, VEGF-B and platelet growth factor (PlGF) to functional receptors [9]. Mice conditioned to over-express podocyte specific VEGF were albuminuric and developed glomerular disease [10], [11]. However, recently, Eremina et al delineated VEGF-A's fundamental role in forming and maintaining the endothelial cell function and a glomerular filtration barrier [12]. The plausible reasoning of this discrepancy of VEGF-VEGFRs signaling in diabetic nephropathy may be attributable to the fact that human and animal diabetic nephropathy progress at different rates and that they are inconsistent in their reproducing renal disease. There may be unknown mechanisms that keep VEGF levels within a narrow range of normal value in order to maintain renal function and glomerular homeostasis [13]. The recent data suggest that VEGF-dependent VEGFR2 activation recruits PI3K, which in turn activates Akt thereby directly phosphorylating eNOS causing increased NO production. The Akt-eNOS activation is critical for the survival and function of glomerular endothelial cells and podocytes [14], [15]. Paradoxically, prolonged exposure of renal cells to high glucose environment has been shown to inhibit cell proliferation and induce growth arrest or cellular apoptosis [16]. These cellular effects are caused by the activation of an intracellular PI3K/Akt pathway and their downstream pathway activation of mTOR-cytochrome C-caspases [17]. Moreover, providing either a precursor for NO production or a stable precursor of tetrahydrobiopterin significantly improved the function of glomerular endothelial cells (GECs) and decreased progression of glomerular injury in type 2 *db/db* mice [18]. We also have found that the treatment with dRK6 that inhibits VEGF-A-VEGFR2 binding did not alter hyperglycemia, but resulted in a severe form of renal damage in type 2 *db/db* mice, regardless of the treatment duration and brought about the suppression of the eNOS-nitric oxide (NO) axis, followed by the uncoupling of VEGF from eNOS-NO [18]. These findings support a notion that it is eNOS dysfunction, not PI3K-Akt activation, that has a major role in pathogenesis of diabetic nephropathy in type 2 diabetes [18], [19].

Most of the cellular actions of VEGF in diabetic nephropathy are predominantly regulated by pan-VEGFR tyrosine kinase or VEGFR2. In spite of its essential roles in embryogenesis, the function of VEGFR1 is poorly understood, especially in diabetic nephropathy. Therefore, the current study investigated the changes and roles of VEGFR1 and their downstream signaling of PI3K-Akt, FoxOs and eNOS-NO in type 2 diabetes and in cultured human glomerular endothelial cells (HGECs) using a VEGFR1-selective hexapeptide, Gly-Asn-Gln-Trp-Phe-Ile (GNQWFI). GNQWFI is a non-immunogenic antagonistic peptide that specifically binds to VEGFR1 to exert inhibitory action with ligands as VEGF-A, VEGF-B, and placental growth factor (PlGF) [20] thereby hindering VEGF-induced endothelial cell migration and angiogenesis both *in vivo* and *in vitro* [20]. We also used small interfering (si) RNA targeted to VEGFR1 to study the role of VEGFR1 in HGECs in high-glucose media.

## Methods

### Experimental methods

All experiments were performed in accordance with our institutional animal care guidelines of the Catholic University of Korea, Seoul, and all the procedures complied with the *Guide for the Care and Use of Laboratory Animals* (National Institutes of Health Publication No. 85-23, revised 1996). Six-week-old male C57BLKS/J *db/m* and *db/db* mice were purchased from Jackson Laboratories (Bar Harbor, ME). Non-diabetic *db/m* and diabetic *db/db* (n = 6, respectively) were used as the controls. As for the GNQWFI (VEGFR1 inhibitor) treatment group, *db/m* and *db/db* mice were divided into *db/m* cont, *db/m*-VEGFR1, *db/db* cont, and *db/db*-VEGFR1 (n = 8, respectively). All the treated mice received three times per week, a subcutaneous injection of 100 µg GNQWFI, according to the previous study [20] that was dissolved in phosphate buffer saline starting at 8 weeks of age for 12 weeks. At week 20, all animals were anesthetized and sacrificed.

### Ethics Statement

All procedure of animal research were provided in accordance with the Laboratory Animals Welfare Act, the Guide for the Care and Use of Laboratory Animals and the Guidelines and Policies for Rodent experiment provided by the IACUC(Institutional Animal Care and Use Committee) in school of medicine, The Catholic University of Korea. (Approval number:Catholic University St.Vincent Hospital 09-06-01)

### Assessment of renal function

At week 20, the animals were housed in metabolic cages (Nalgene, Rochester, NY) for 24-h to collect urine for subsequent measurements of the albumin concentrations by an immunoassay (Bayer, Elkhart, IN). Plasma and urine creatinine concentrations were measured using a HPLC (Beckman Instruments, Fullerton, CA).

### Histology

We performed immunoflourescene for VEGFR1 and –R2 and immunohistochemistry for TGF-β1, type IV Col, nephrin, osteopontin, WT-1, F4/80, PECAM-1, and Ki-67, and a TUNEL assay. Briefly, small blocks of kidney were immediately fixed in 10% buffered formalin for 24 h before being embedded in paraffin. Four-micrometer-thick sections were deparaffinized, hydrated in ethanol and treated with an antigen unmasking solution (consisted of 10 mM Sodium citrate buffer, pH 6.0) and then washed with phosphate buffered saline. The sections were incubated with 3% H_2_O_2_ in methanol to block the endogenous peroxidase activity. Non-specific binding was blocked with 2.5% normal horse serum in phosphate buffer solution. The sections were incubated overnight with anti-TGF-β1 (R&D Systems, Minneapolis, MN), anti-Col IV (Biodesign International, Saco, ME), anti-nephrin (United States Biological, Swampscott, MA, USA), anti-VEGFR1 (Epitomics, Burlingame) and –R2 (Cell Signaling Technology, Danvers, MA), anti-osteopontin (MPIIIb10, the Developmental Studies Hybridoma Bank, University of Iowa, IA), WT-1 (Santa Cruz Biotechnology, Santa Cruz, CA), anti-F4/80 (Serotek, Oxford, UK), anti-PECAM-1 (BD Bioscience, San Diego, CA), Ki-67 (Abcam, Cambridge, UK) and 8-OH-dG (CosmoBio, Tokyo, Japan) in a humidified chamber at 4°C. The antibodies were localized with a peroxidase-conjugated secondary antibody using the Vector Impress kit (Vector Laboratories, Burlingame, CA) and 3,3-diamninobenzidine substrate solution. The sections were then dehydrated in ethanol, cleared in xylene, and mounted without counterstaining. All of these sections were examined in a blinded manner using light microscopy (Olympus BX-50, Olympus Optical, Tokyo, Japan). For the quantification of the proportional areas of staining, approximately 20 views (×400 magnification) were randomly located in the renal cortex and the corticomedullary junction of each slide (Scion Image Beta 4.0.2, Frederik, MD). The proportion of apoptotic cells were also determined using ApopTag In Situ Apoptosis Detection Kits (Chemicon-Millipore, Billerica, MA), based on a terminal deoxynucleotidyl transferase-mediated dUTP nick-end labeling (TUNEL) assay. The TUNEL reaction was determined in the whole glomeruli biopsy under ×400 magnification.

### Western blotting in the kidney

The total proteins of the kidney tissues were extracted with a Pro-Prep Protein Extraction Solution (Intron Biotechnology, Gyeonggi-Do, Korea) according to the manufacturer's instructions. Western blot analysis was performed to further confirm the responses using antibodies that recognize the specific epitope. The proteins were separated by SDS-PAGE, transferred to nitrocellulose membranes, and detected with the following antibodies: PI3k (BD Biosciences, Franklin Lakes, NJ), total-Akt (Cell Signaling Technology, Danvers, MA), phospho-Ser^473^ Akt (Cell Signaling Technology, Danvers, MA), active caspase-3 (Santa Cruz Biotechnology, Santa Cruz, CA), Bcl-2 (Santa Cruz Biotechnology, Santa Cruz, CA), Bax (Santa Cruz Biotechnology, Santa Cruz, CA), Bim (Cell Signaling Technology, Danvers, MA), superoxide dismutase (SOD)1 (Assay Designs, Ann Arbor, MI, USA), SOD2 (Abcam, Cambridge, UK), and β-actin (Sigma-Aldrich, St Louis, MO). After washing, the membrane was incubated with anti-mouse IgG or anti-rabbit IgG, HRP-linked secondary antibody (Cell Signaling Technology, Danvers, MA) or anti-goat IgG HRP-perosidase antibody that was produced in a rabbit (Sigma-Aldrich, St Louis, MO). The blot was then developed using the ECL Plus detection kit (Amersham International, Bucks, UK) to produce a chemiluminescence signal, which was captured on X-ray film. The density of each band was quantified with Quantity One software (Bio-Rad Laboratory, Hercules, CA).

### Measurement of Phosphatidylinositol 3-Kinase enzyme activity in kidney tissue

Kidney tissues were homogenized in the presence of protease inhibitors to obtain extracts of kidney proteins. Protein concentrations were determined using Bradford reagent (Bio-Rad, Hercules, CA) The amount of phosphatidyl inositol-3,4,5-triphosphate (PIP3) produced was quantified by PIP3 competition enzyme immunoassays according to the manufacturer's protocol. (Echelon, Inc., Salt Lake City, UT) The enzyme activity was expressed as picomoles of PIP3 produced by kidney tissue extracts containing equal concentrations (1 mg) of total protein.

### Real-time PCR in the kidney

The VEGFR1 and −2 mRNA levels were determined by quantitative real time-PCR, using SYBR® Premix Ex TaqTM (TaKaRa Bio, Shiga, Japan) with specific primers (Table 1). We used 18 s rRNA as an internal control.

**Table 1:** Primers for Quantitative Real-Time PCR.

Gene	Forward	Reverse
*VEGF-R1*	5′-ACATGGGACAGTAGGAGA-3′	5′-ACGGAGGTGTTGAAAGAC-3′
*18s rRNA*	5′-CGCGGTTCTATTTTGTTGGT-3′	5′-AGTCGGCATCGTTTATGGTC-3′

### 24-h urinary 8-hydroxy-deoxyguanosine (8-OH-dG) and nitrates

To evaluate oxidative DNA damage and lipid peroxidation, we measured the 24-h urinary 8-hydroxy-deoxyguanosine (8-OH-dG; OXIS Health Products, Inc., Portland, OR). The total urinary NO3^−^ and NO2^−^ excretion was quantified using the Nitric Oxide Assay Kit (Bio Vision, Mountain View, CA).

### Western Blotting, TUNEL Assay, NOx Levels in the HGECs Culture

HGECs were purchased from Anigio-Proteomie (Boston, MA) and subcultured in Endo-growth media (Angio-Proteomie, Boston, MA). Apoptosis was quantified using the *in situ* cell death detection kit by TUNEL assay (Chemicon-Millipore, Billerica, MA). After treatment with different concentrations of media with D-glucose (5 mM/L D-glucose; low-glucose, 30 mM/L D-glucose; high-glucose, and D-glucose [5 mM/L]+D-mannitol [25 mM/L]; osmotic control) including 10^−6^ M GNQWFI (which showed a nearly complete inhibition of VEGF/VEGFR1 interaction) [20] for 72 hr, the number of TUNEL-positive cells was counted in 10 randomly chosen fields at a magnification of 400×. Western blot analysis was performed for PI3k, total-Akt, phospho-Ser^473^ Akt, total eNOS, phospho-Ser^1177^ eNOS, SOD1, SOD2 and β-actin with specific antibodies. siRNA targeting VEGFR1 and scrambled siRNA (siRNA control; Bioneer, Deajeon, Korea) were complexed with transfection reagent (Lipofectamin 2000; Invitrogen, Carlsbad, CA) in low-glucose media according to the manufacturer's instructions.

### Statistical analysis

The data is expressed as mean ± standard deviation (SD). Differences between the groups were examined for statistical significance using ANOVA with Bonferroni correction using SPSS version 11.5 (SPSS, Chicago, IL). A *p* value<0.05 was considered to be a statistically significant difference. Non-normally distributed data were analyzed by the Mann-Whitney *U*-test.

## Results

### Physical and biochemical characteristics in *db/m* and *db/db* mice with VEGFR1 Inhibition

Body weights, kidney weights, blood glucose concentrations, and HbA1c were significantly higher in the diabetic *db/db* mice groups than in the non-diabetic *db/m* mice groups, regardless of VEGFR1 inhibition (Table 2). However, there were no differences in those levels among any of the *db/db* or *db/m* study groups. There was no significant difference in serum creatinine level among all any of the study groups. Mice in the *db/db* group had an increased likelihood of albuminuria and greater urine volume compared to *db/m* mice. Decreased urine volume in VEGFR1 inhibition is consistent with previous study, which showed VEGF antibody treatment abolished renal hyperfilatration in *db/db* mice [22]. Interestingly, despite having the same degree of hyperglycemia, the *db/db*-VEGFR1 mice were more likely to have albuminuria compared to the *db/db* cont group. However, no such differences were detected in the *db/m* mice groups (Table 2).

**Table 2:** Biochemical and physical characteristics of all study groups.

	*db/m*		*db/db*	
	cont	VEGFR1	cont	VEGFR1
Body wt (gm)	32±2	31±3	46±5#	44±6#
Kidney wt (gm)	0.19±0.03	1.19±0.01	0.24±0.02*	0.23±0.03*
FBS (mmol/l)	10.3±0.6	9.3±0.7	36.2±10.7#	33.8±1.7#
HbA1c (%) (mmol/mol)	4.6±0.3 (50.3±3.3)	4.7±0.2 (51.4±2.2)	11.4±1.3# (101±13)	11.2±1.0# (99±12)
Urine volume(ml)	1.0±0.4	1.2±0.4	14.7±4.6#	6.0±3.3*
24 hr urinary albumin (µg)	8.6±6.6	6.9±1.8	151.1±21.1*	287.0±47.6**
Serum Cr (µmmol/l)	7.9±0.8	7.9±0.8	7.0±0.8	7.9±1.7
Systolic BP (mmHg)	98.6±4.5	96.4±5.5	101.1±5.2	99.5±4.9

Abbreviations: Ccr; creatinine clearance, Cr; creatinine, FBS; fasting blood sugar.

*P<0.05,

**P<0.01,

# *P*<0.001 compared to other group.

### Renal expression of VEGFR1 and -R2 mRNA and protein in *db/m* and *db/db* mice

**Figure 1:**
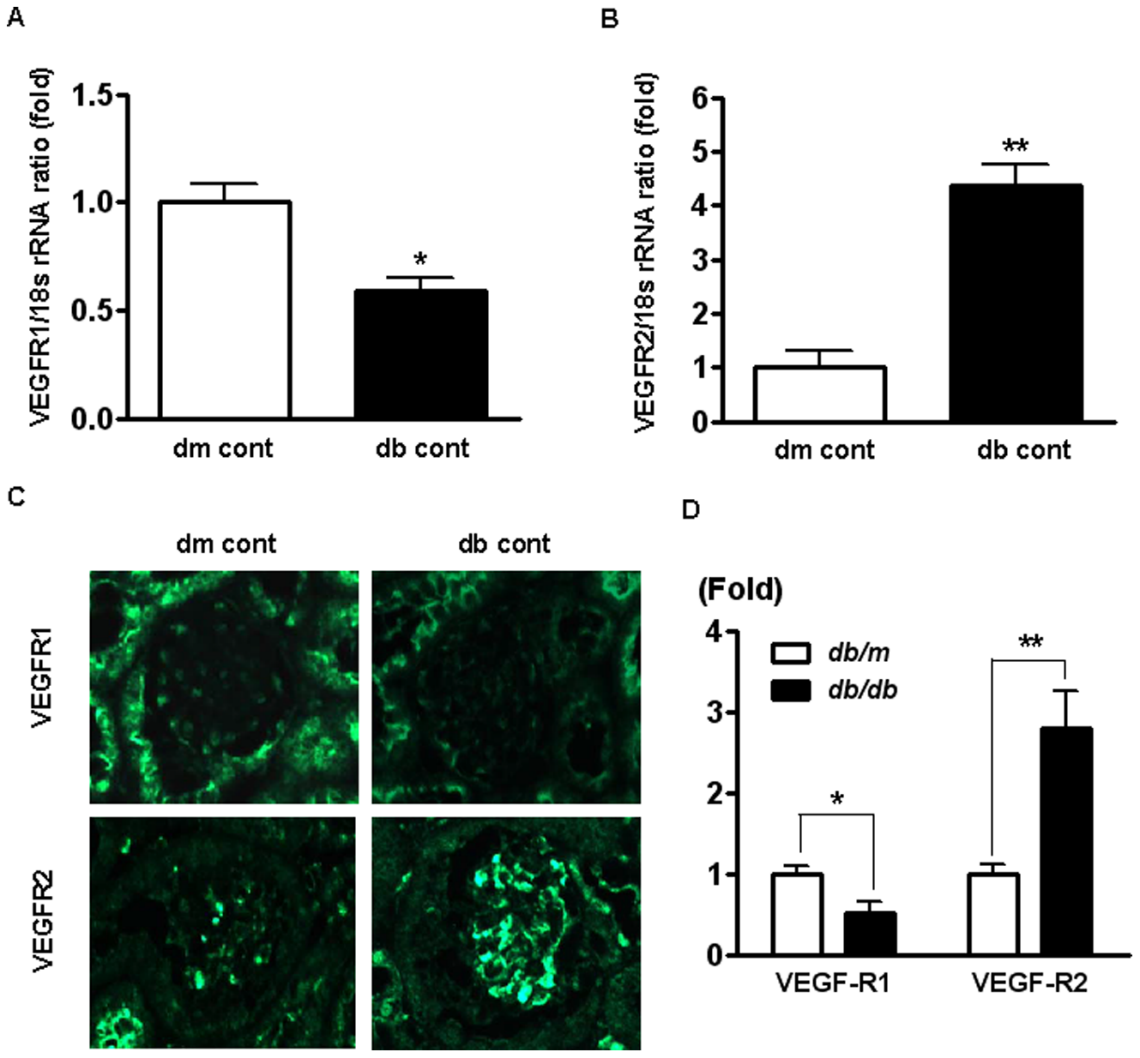
VEGFR1 and R2 mRNA and protein expression in the renal cortex of the *db/m* and *db/db* mice at 12 weeks of the study. Representative real-time RT-PCR results. *P<0.05, **P<0.01 compared with db/m mice. (C) Representative and immunohistochemistry (×400). Representative immunohistochemical staining for VEGFR1 and -R2 and quantitative analyses of the immunohistochemical staining are shown.

Renal cortical expression of VEGFR1 and -R2 was determined by real-time PCR. The expression of VEGFR1 mRNA was decreased by 40% in *db/db* mice compared to *db/m* mice (Figure 1). In contrast, the expression of VEGFR2 mRNA increased 4.2-fold in *db/db* mice compared to *db/m*. Protein levels were measured using immunohistochemistry, and were consistent with changes in mRNA expression: VEGFR1 decreased by 48% in *db/db* mice compared to *db/m* mice.

### Effects of VEGFR1 inhibition on the renal phenotypes, TGF-β1, type IV collagen (Col IV), and F4/80

**Figure 2:**
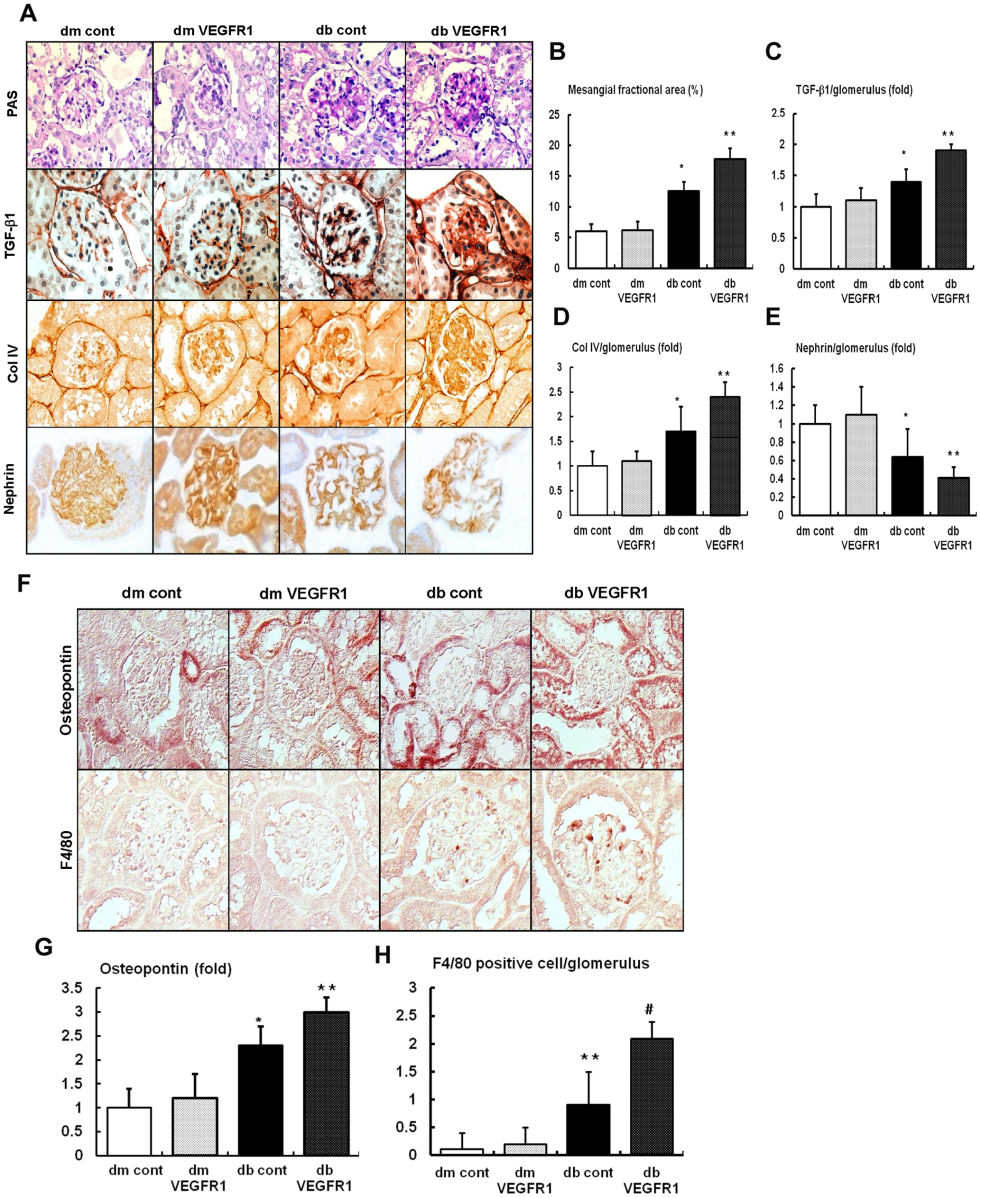
Renal glomerular extracellular matrix, nephrin expression and inflammation in the renal cortex of *db/m* and *db/db* mice with or without VEGFR1 inhibition. (A to E) Representative Periodic Acid Schiff (PAS) stain and immunohistochemical staining for TGF-β1, Col IV and nephrin and quantitative analyses of the mesangial fractional area and the immunohistochemical staining are shown. (F to H) Representative immunohistochemical staining for osteopontin, F4/80 positive cell and the quantitative analyses of the immunohistochemical staining results are shown. *P<0.05, **P<0.01 and ^#^ *P*<0.001 compared with *db/m* mice groups.

There were no differences in the fractional mesangial area, TGF-β1, Col-IV and nephrin among the *db/m* mice groups regardless of VEGFR1 inhibition (Figure 2). In contrast, there was a 1.9-fold increase in mesangial area expansion in the *db/db* cont mice as compared to that of the *db/m* cont mice (*P*<0.01). Consistent with the change in the mesangial fractional area, the expression of the pro-fibrotic growth factor TGF-β1, which is associated with extracellular matrix Col IV and fragmentation of nephrin, increased in the *db/db*-VEGFR1 compared to *db/db* cont mice. Moreover, the expression of osteopontin, as a chemoattractant, increased significantly in the *db/db* cont and *db/db*-VEGFR1 mice (Figure 2). As demonstrated by the immunohistochemical analysis, all of the diabetes-induced renal phenotypic changes and inflammation seen in the *db/db* cont were aggravated in the *db/db*-VEGFR1 mice (Figure 2). In contrast, diabetes-induced renal phenotype changes related to VEGFR1 inhibition were not observed in *db/m* mice.

### Effects of VEGFR1 inhibition on the glomerular cells

**Figure 3:**
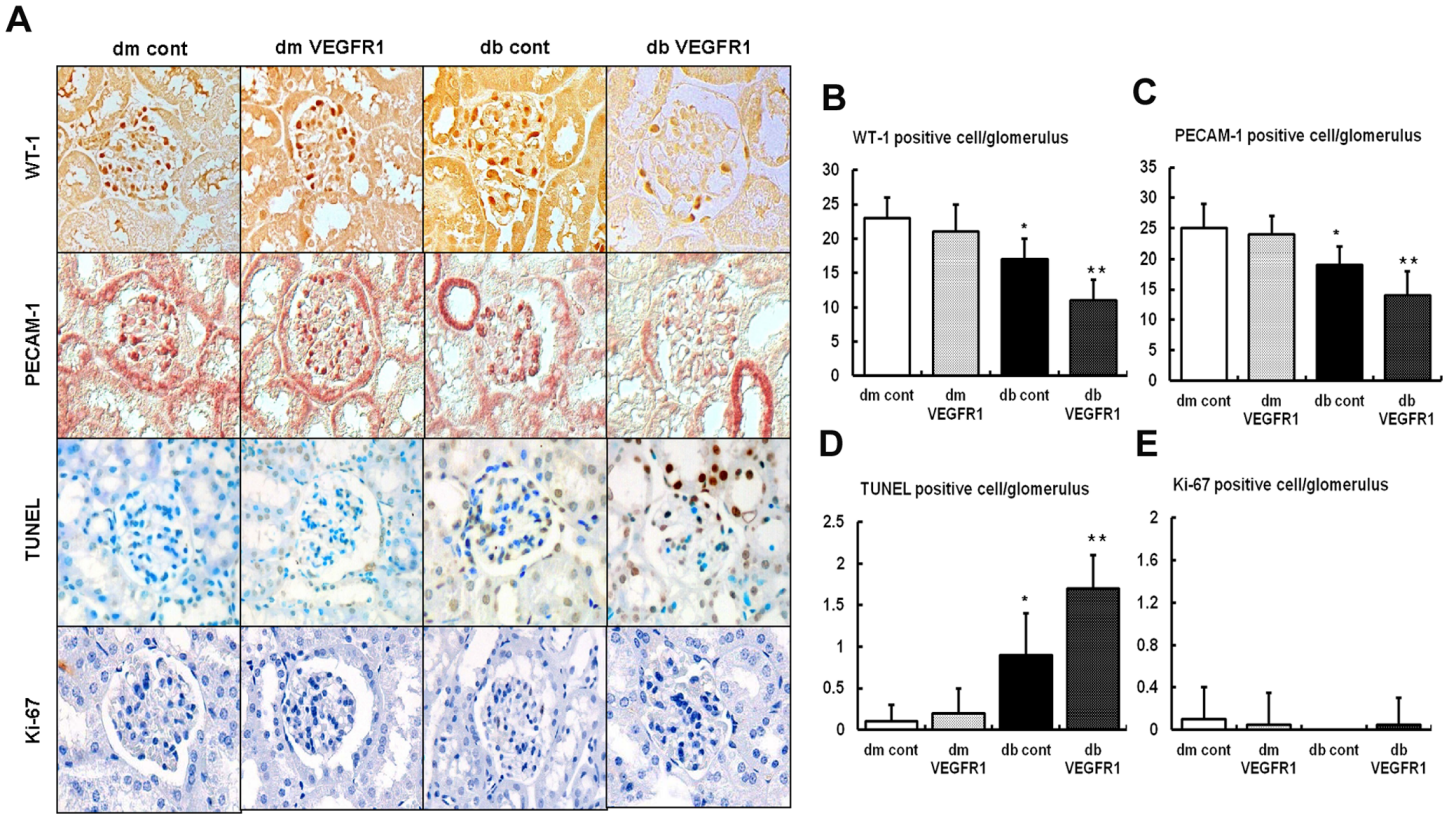
Renal glomerular cell expression in the renal cortex of *db/m* and *db/db* mice with or without VEGFR1 inhibition. Representative WT-1 positive cell (podocyte marker), PECAM-1 (endothelial cell marker), TUNEL-positive cell (apoptotic cell marker), and Ki-67 positive cell (cell proliferation marker). And, the quantitative analyses of the immunohistochemical staining results are shown. *P<0.05, **P<0.01 compared with *db/m* mice groups.

Decreased quantities of cells in the glomerulus, especially podocytes and glomerular endothelial cells, are considered one of main causes of diabetic nephropathy [21],[22]. Therefore, this study determined the number of the glomerular cells associated with VEGFR1 inhibition. There were no differences in the number of WT-1, PECAM-1, TUNEL, or Ki-67 positive cell in the *db/m* mice groups (Figure 3). In contrast, there were significant decreases in the number of WT-1 and PECAM-1 positive cells in the *db/db* cont, which showed particularly large decreases in the *db/db*-VEGFR1 mice and were associated with an increased number of TUNEL positive cells. There was no difference in the number of Ki-67 positive cells among any of the study groups, suggesting that there is no proliferation of glomerular cells. Altogether, apoptosis plays a major role in the depletion of podocytes and glomerular endothelial cells as they relate to VEGFR1 inhibition in type 2 diabetes.

### Renal expressions of the PI3K-Akt-FoxO3a-eNOS signaling pathways and SOD1 and SOD2

**Figure 4:**
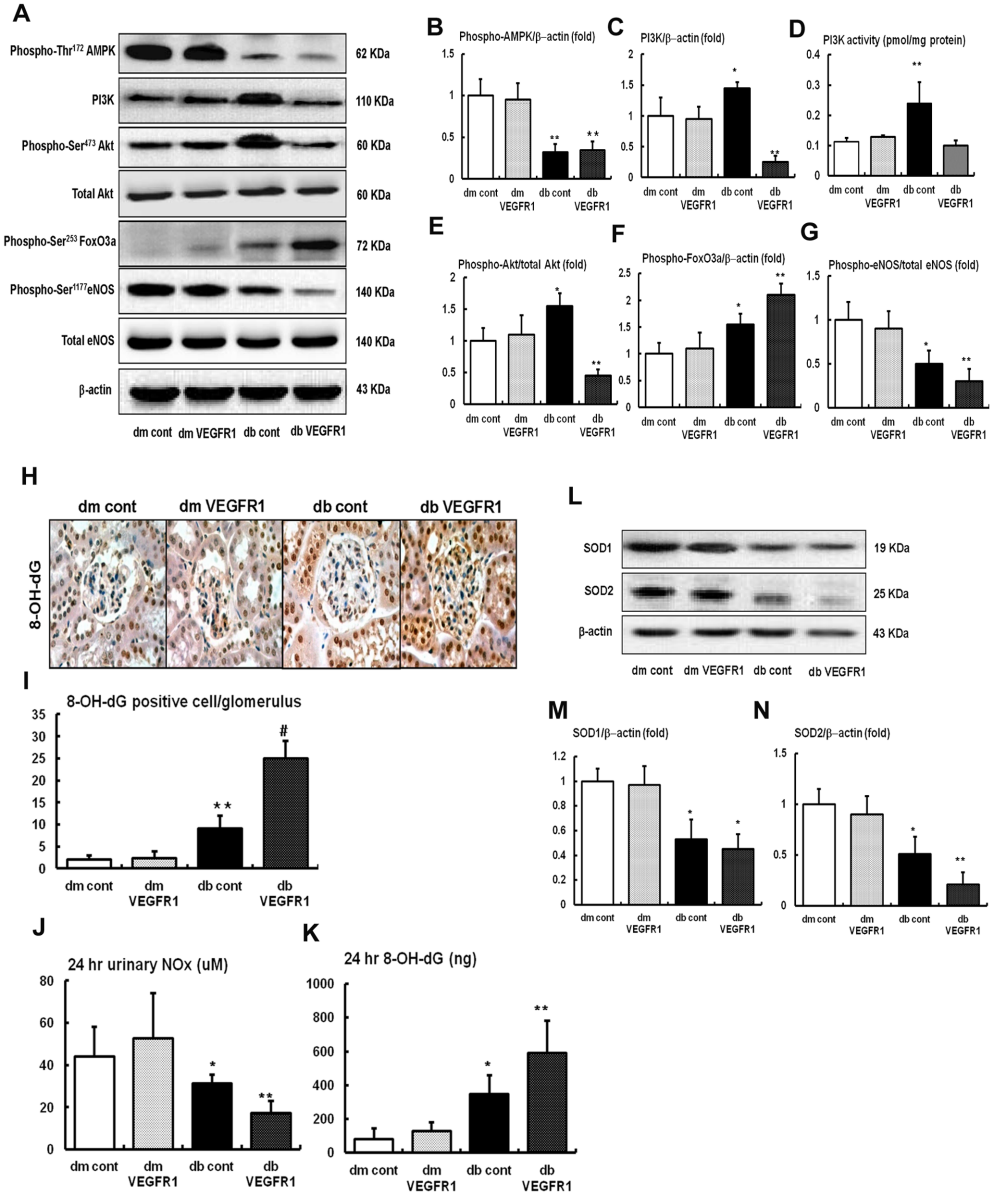
Phospho-Thr^172^ AMPK, total AMPK, PI3K level and activity, phospho-Ser^473^ Akt, total Akt, phospho-Ser^453^ FoxO3a, phospho-Ser^1177^ eNOS, total eNOS and SOD1, SOD2 expressions in the renal cortex and 24-h urinary NOx, 8-OH-dG concentrations of the *db/m* and *db/db* mice with or without VEGFR1 inhibition. (A to G) Representative Western blot analysis of the phospho-Thr^172^ AMPK, total AMPK, PI3K, phospho-Ser^473^ Akt, total Akt, phospho-Ser^453^ FoxO3a, phospho-Ser^1177^ eNOS, total eNOS and β-actin expression and the quantitative analyses of the results are shown. (L to N) Representative Western blot analysis of the SOD1, SOD2 and β-actin expression and the quantitative analyses of the results are shown. (H to K) Representative 24-h urinary NOx, 8-OH-dG concentrations of the results are shown. *P<0.05, **P<0.01 compared with *db/m* mice groups.

Intra-renal phospho-Thr^172^ AMPK, phosphoinositide-3-knase (PI3K), phospho-Ser^473^ Akt, and phospho-Ser^1173^ eNOS levels were determined using Western blot analysis. We also measured PI3K activity by ELISA. There were no differences in phospho-Thr^172^ AMPK, PI3K- phospho-Ser^473^ Akt and phospho-Ser^453^ FoxO3a signaling among the *db/m* mice groups regardless of VEGF-R inhibition (Figure 4). Phospho-Thr^172^ AMPK levels were decreased in *db/db* mice groups, which were not changed after VEGFR1 inhibition. However, PI3K levels and activity, the phospho-Ser^473^/total-Akt ratio and phospho-Ser^453^ FoxO3a increased significantly in *db/db* cont mice compared to *db/m* mice groups (*P*<0.05) (Figure 4). In contrast, a markedly suppression of PI3K levels and activity and pAkt and paradoxically up-regulation of phospho-Ser^453^ FoxO3a expression in the kidney of *db/db*-VEGFR1 mice were noted compared to those of *db/db* cont mice. Because PI3K activation and the phosphorylation of Akt and FoxO3a signaling serve as a survival signal associated with downstream regulator phosphorylation of eNOS, changes in phospho-Ser^1173^ eNOS were also examined. Decreased phospho-Ser^1173^ eNOS expression was also noted in *db/db* cont mice compared to *db/m* mice (*P*<0.05) (Figure 4). Consistent with PI3K-pAkt changes in *db/db*-VEGFR1 mice, the inhibition of VEGFR1 with GNQWFI markedly decreased the expression of phospho-Ser^1173^ eNOS in *db/db* mice, which resulted in a decrease in NOx and an increase in 24 hr 8-OH-dG, reflecting an increase in oxidative stress in the kidney (Figure 4). SOD1 and SOD2 levels were lower in *db/db* mice than in *db/m* mice groups. In contrast, SOD2 levels were significantly decreased in *db/db*-VEGFR1 mice compared to *db/db* cont mice (Figure 4).

### Renal expression of pro-apoptotic Bim, Bax, anti-apoptotic Bcl-2 and cleaved caspase-3 expressions

**Figure 5:**
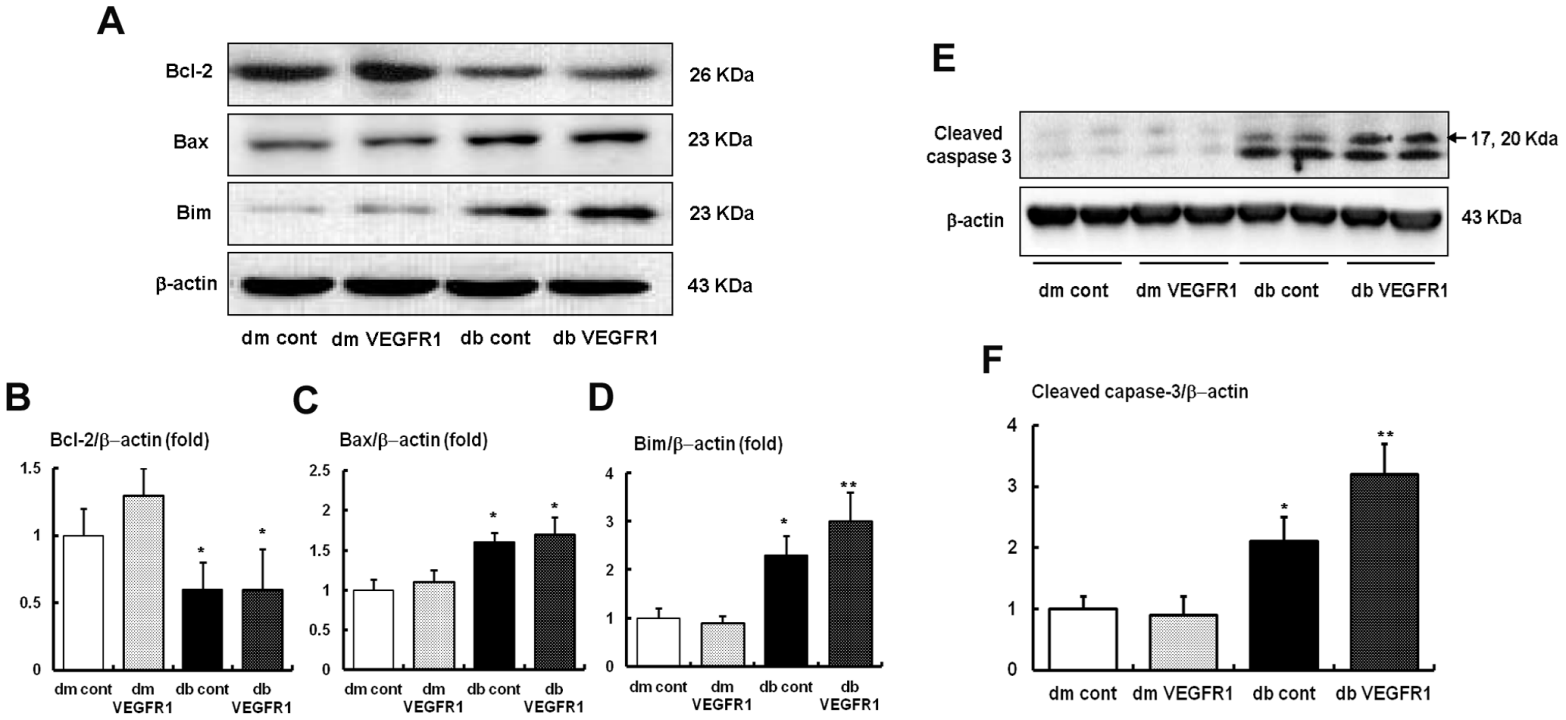
Proapoptotic Bim, Bax and cleaved caspase-3 and antiapoptotic Bcl-2 expressions in the renal cortex of the *db/m* and *db/db* mice with or without VEGFR1 inhibition. (A to D) Representative Western blot analysis of the Bim, Bax, Bcl-2 and β-actin expressions and the quantitative analyses of the results are shown. (E and F) Representative Western blot analysis of the cleaved caspase-3 and β-actin expressions and the quantitative analyses of the results of cleaved caspase-3 are shown. *P<0.05 and **P<0.01compared with *db/m* mice groups.

It is well known that FoxOs activation and eNOS phosphorylation has anti-stress and anti-apoptotic activities through the enhancing Bcl-2 activity and the downregulation of pro-apoptotic Bim activity [21]. In accordance with the changes of eNOS, Bim, Bax and Bcl-2 levels as measured on Western blot analysis increased and decreased, respectively, in *db/db* cont mice compared to *db/m* mice. In contrast to the levels of the Bcl-2 and Bax protein, which did not changed with VEGFR1 inhibition, the expression of Bim protein increased further in *db/db*-VEGFR1 mice compared to *db/db* cont mice. Consequently, the Bcl-2/Bim ratio decreased significantly in *db/db* mice, resulting in an increase in cleaved caspase-3, which is a critical executioner of apoptosis (*P*<0.01) (Figures 5). VEGFR1 inhibition in *db/db* mice ultimately increased cleaved caspase-3 expression (Figure 5).

### *In vitro* studies

**Figure 6:**
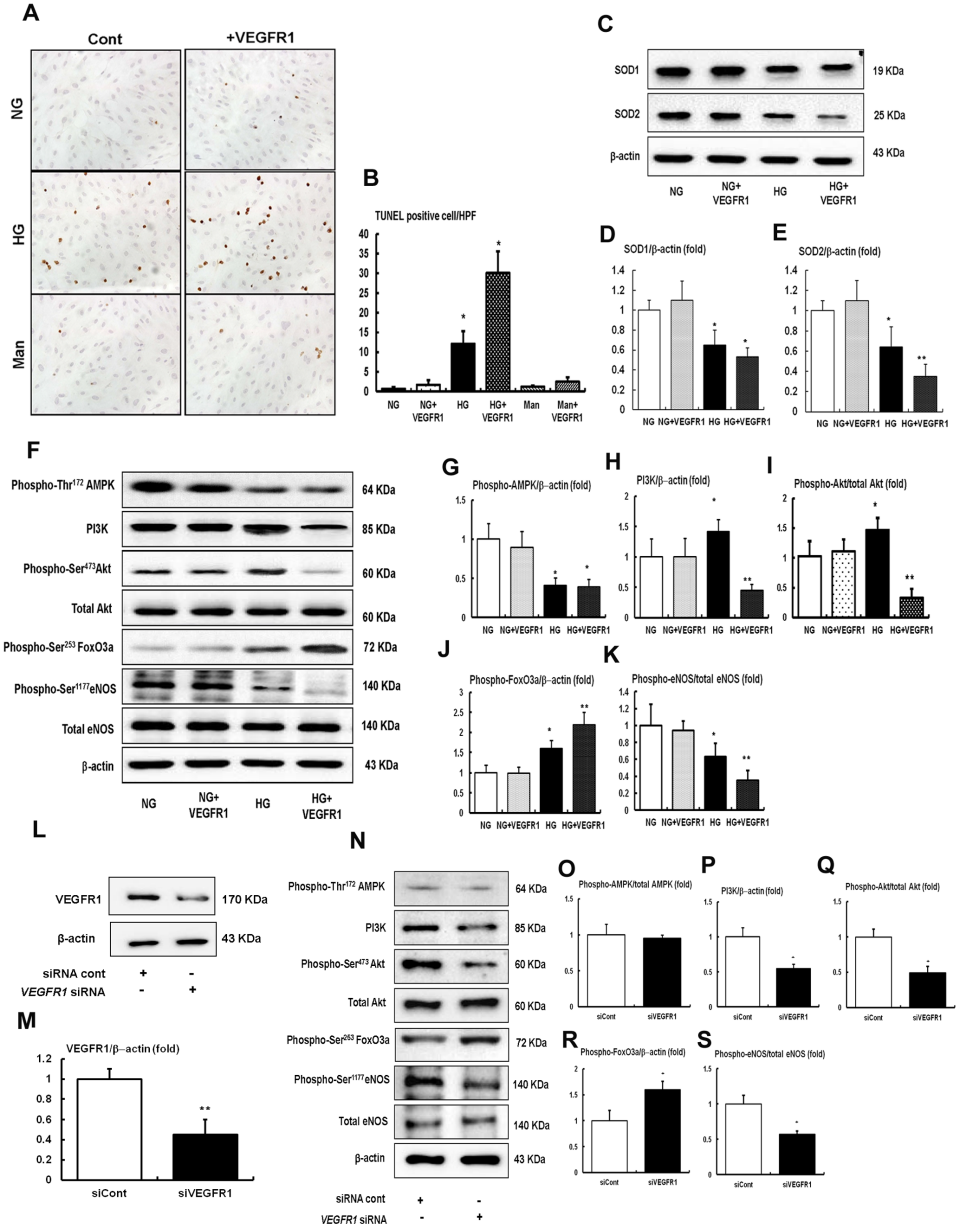
The effect of VEGFR1 inhibition on apoptosis, intracellular PI3K-Akt-FoxO3a-eNOS signaling and SODs in HGECs cultured in low glucose (5 mmol/L of D-glucose) or high glucose (30 mmol/l of D-glucose). (A and B) Representative pictures of TUNEL-positive HGECs (original magnification ×400) and the quantitative analyses of the results are shown. *P<0.05, **P<0.01 compared with normal glucose or normal glucose with VEGFR1 inhibition groups. (C to E) SOD1 and SOD2 expressions in the HGECs. Representative Western blot analysis of the SOD1 and SOD2 and the quantitative analyses of the results are shown. *P<0.05, **P<0.01 compared with normal glucose or normal glucose with VEGFR1 inhibition. (F to K) Representative Western blot analysis of the phospho-Thr^172^ AMPK, total AMPK, PI3K, phospho-Ser^473^ Akt, total Akt, phospho-Ser^453^ FoxO3a, phospho-Ser^1177^ eNOS, total eNOS and β-actin expressions in HGECs and the quantitative analyses of the results are shown. *P<0.05 and **P<0.01 compared with normal glucose or normal glucose with VEGFR1 inhibition. n = 4. (L and M) The cultured HGECs were transfected with 50 nM control siRNA (siRNA cont), 50 nM of VEGFR1 (VEGFR1 siRNA) in low-glucose media. Approximately 48 h stimulation, the expressions of the VEGFR1 were analyzed. **P<0.01 compared with siRNA control. n = 4. (N to S) Representative Western blot analysis of the phospho-Thr^172^ AMPK, total AMPK, PI3K, phospho-Ser^473^ Akt, total Akt, phospho-Ser^453^ FoxO3a, phospho-Ser^1177^ eNOS, total eNOS and β-actin expressions in HGECs with or without VEGFR1 siRNA and the quantitative analyses of the results are shown. *P<0.05 and **P<0.01 compared with siRNA cont. n = 4.

We evaluated the effects of VEGFR1 inhibition on high glucose-induced oxidative stress and apoptosis related to the PI3K-Akt-FoxO3a-eNOS signaling in HGECs. VEGFR1 inhibition in high-glucose media increased the number of TUNEL positive HGECs compared with high-glucose media alone (30 mmol/L of D-glucose), which was associated with decreased SOD1 and SOD2 expression (Figure 6) when compared to high-glucose media alone. High glucose-induced HGECs apoptosis and oxidative stress related to a decrease in FoxO3a activity and subsequent decrease in eNOS-NO expression in spite of high PI3K-pAkt signaling (Figure 6), without changes of phospho-Thr^172^-AMPK levels. In contrast, compared with high-glucose media, the inhibition of either VEGFR1 in low glucose (5 mmol/L of D-glucose) and osmotic control mannitol (25 mmol/L mannitol plus 5 mmol/L D-glucose; data not shown) did not affect HGECs apoptosis or SOD1 and SOD2 expressions, nor did it impact PI3K-Akt-FoxO3a-eNOS signaling (Figures 6). Additional experiments were performed using siRNAs of VEGFR1 in cultured HGECs to confirm that the pro-apoptotic effect of VEGFR1 inhibition is PI3K-Akt-FoxO3a-eNOS-dependent. Transfection with VEGFR1 siRNA suppressed PI3K expression and phosphorylation of Akt as well as inactivation of FoxO3a-eNOS signaling, when compared to the siRNA control (Figure 6). Consistent with in vivo study, there was no change in phospho-Thr^172^-AMPK (Figure 6).

## Discussion

Diabetes suppressed VEGFR1 and increased VEGFR2 expressions in the kidneys. The prominent features in db/db mice with VEGFR1 inhibition were albuminuria, glomerular mesangial matrix expansion and inflammatory cell infiltration, and profibrotic TGF-β1 expression. VEGFR1 inhibition also resulted in the increase in both the number of apoptotic glomerular cells and 24-h urinary 8-OH-deoxyguanosine concentrations compared to that of *db/db* control mice. All these changes were associated with the suppression of diabetes-induced upregulation of renal PI3K-Akt phosphorylation and further suppression of diabetes-induced inactivation of FoxO3a and eNOS-NO pathway. In contrast, VEGFR1 blockade-induced renal phenotypes were not observed in *db/m* mice groups. In HGECs, high-glucose media treated with VEGFR1 inhibitor induced more apoptotic cell death and oxidative stress than did high-glucose media, and this induction was associated with a suppression of the PI3K-Akt-eNOS signaling resembling in *db/db* mice, finally resulting in oxidative stress. Therefore, the selective blockade of VEGFR1 in *db/db* mice caused severe renal injury due to the imbalance among the VEGF-VEGFR1, PI3K-Akt-FoxO3a pathway and eNOS-NO, which is duly associated with apoptotic damage that is induced by enhanced oxidative stress.

Increased VEGFR2 gene and protein expression has been well demonstrated in the kidneys, glomerulus in particular, of diabetic rats exposed to high glucose environment for either short-term [23], [24] or long-term diabetic rats [15]. Meanwhile, VEGFR1 mRNA and protein levels in the kidney of *db/db* mice decreased in comparison to those in the kidneys of *db/m* mice. Regarding VEGFR1, however, it is not fully understood as to how it works in diabetic nephropathy. It has been studied that a dominant form of VEGFR1 in the kidney is soluble one, which lacks an intracellular signaling domain [25]. Other studies have suggested that there is only minimal or zero expression of VEGFR1 in the kidney [26]–[28], indicating that this protein has neither initiated a VEGF-A signal transduction pathway nor is not primarily responsible for biological actions of VEGF in the adults [29], [30]. VEGF-A can also time-dependently induce phosphorylation of VEGFR1 in cultured glomerular endothelial cell [31]. The current study demonstrated that VEGFR1 is expressed in glomerular and tubular cells, including HGECs, and that VEGFR1 inhibition also caused severe renal damage in the way VEGFR2 inhibition had done to diabetic *db/db* mice.

Most of the cellular actions of VEGF-A are mediated by VEGFR2. Activation of its downstream signaling events, includes tyrosine phosphorylation of insulin receptor substrate (IRS)-1 and PI3K/Akt signaling, which converge on eNOS phosphorylation on Ser^1173^ [28]. eNOS is known to regulate angiogenesis [30], [31] and has healing effects of damaged endothelial cells in thrombotic microangiopathy and remnant kidney models of renal disease related to the oxidative stress [32]–[34]. While NO generated by eNOS inhibits adhesion of leukocytes to the endothelium, NO dysregulation may be involved in pathogenesis of glomerulonephritis [35]. These observations show that VEGF is essential in glomerular endothelial integrity. In contrast, growth factors may have a pathogenic role in diabetic nephropathy. Prolonged exposure of renal cells to high glucose environment increased PI3K and Akt phosphorylation in association with mTOR activation, inactivation of Bcl-2, increased cytosolic cytochrome C expression, and activation of caspase 3 [16], [36], resulting in the cellular hypertrophy or apoptosis, which was prevented by AMPK activators, such as 5-aminoimidazole-4-carbosamide-1β-riboside (AICAR) and metformin [37]. Moreover, providing either a precursor for NO production or a stable precursor of tetrahydrobiopterin significantly improved the function of glomerular endothelial cells (GECs) while ameliorating the progression of glomerular injury in type 2 *db/db* mice [18]. Recently, our group demonstrated that the treatment with dRK6 that inhibits VEGF-VEGFR2 resulted in a severer form of renal damage in *db/db* mice due to renal dysfunction associated with uncoupling of the Akt/eNOS-NO axis regardless of the treatment duration [19]. The current study added a new finding that selective VEGFR1 inhibition alone could lead to renal damage in *db/db* mice and that irrespective of the types of diabetic animal models, diabetic nephropathy caused by inactivation of the eNOS-NO axis, results from VEGF's uncoupling from eNOS-NO.

Many previous studies have drawn attention to the importance of the PI3K pathway in cell survival. While they engage themselves in rescuing cells such as podocytes, PI3K and its downstream Akt/PKB phosphorylation also deliver an antiapoptotic signal induced by various stimuli by regulation of bcl-2 family [21], [37]–[39]. In contrast, the current study suggests that elevated PI3K-Akt phosphorylation in *db/db* mice works deleteriously while mediating the development and progression of diabetic nephropathy in cases without a subsequent activation of the eNOS-NO axis. In addition, depressed VEGFR1 in *db/db* mice had made even more suppression in the eNOS-NO axis by GNQWFI while inducing more oxidative stress in the kidney. We cautiously reason that an increase in PI3K activity and Akt phosphorylation may not prevent cellular damage in diabetic condition. Results from this study also demonstrated that VEGFR1 inhibition causes a decrease in the number of podocytes, accompanied by disorganized and fragmented nephrin. Altogether, decreased NO availability may play a significant role in the development of advanced lesions of diabetic nephropathy including podocyte damage through disruption of glomerular autoregulation and uncontrolled VEGF-VEGFR action [40]. Recently, it has been reported that exercise-induced VEGF expression is associated with AMPK activation, which would improve angiogenic potential, decrease oxidative stress and ameliorate hypertension [41]. Our results showed that there was a significant decrease in AMPK phosphorylation in *db/db* mice and HGENs in high-glucose media, however, no relationship was noted between VEGFR1 inhibition and AMPK phosphorylation.

Our study has some limitations and attention needed as to the interpretation of urinary NOx and oxidative DNA damage marker as intrarenal NOx activation and oxidative marker, such as 24 hr urinary NOx and 8-OH-dG concentration. Their urinary concentrations also reflect NO system and oxidative stress of the whole body including, but not limited to, the kidney, in case that the whole vasculature is exposed to the treatment of VEGFR1 inhibitor.

In conclusion, selective inhibition of VEGFR1 in type 2 diabetes was associated with deteriorated renal function, proteinuria, glomerulosclerosis, and inflammation of the kidney in relation the increases in glomerular cell apoptosis and oxidative damage. All of these changes in glomerular endothelial cells were associated with the inactivation of renal and endothelial PI3K-Akt-eNOS-NO pathway. The protective role of VEGF-VEGFRs could be predominately dependent on its ability to stimulate NO production in endothelial cells. Therefore, cautions should be exercised on the modulation of VEGF-VEGFRs while VEGFR1 activation may provide a therapeutic modality in type 2 diabetic nephropathy.
